# An Arc-Shaped Piezoelectric Bistable Vibration Energy Harvester: Modeling and Experiments

**DOI:** 10.3390/s18124472

**Published:** 2018-12-17

**Authors:** Xuhui Zhang, Wenjuan Yang, Meng Zuo, Houzhi Tan, Hongwei Fan, Qinghua Mao, Xiang Wan

**Affiliations:** 1 College of Mechanical Engineering, Xi’an University of Science and Technology, Xi’an 710054, China; Zoezuom@163.com (M.Z.); Houzhitanxy@126.com (H.T.); fanhongwei84@163.com (H.F.); maoqhua-1984@163.com (Q.M.); wx@xust.edu.cn (X.W.); 2 Shaanxi Key Laboratory of Mine Electromechanical Equipment Intelligent Monitoring, Xi’an 710054, China

**Keywords:** vibration energy harvesting, bistable, arc-shaped, nonlinear, piezoelectric

## Abstract

In order to improve vibration energy harvesting, this paper designs an arc-shaped piezoelectric bistable vibration energy harvester (ABEH). The bistable configuration is achieved by using magnetic coupling, and the nonlinear magnetic force is calculated. Based on Lagrangian equation, piezoelectric theory, Kirchhoff’s law, etc., a complete theoretical model of the presented ABEH is built. The influence of the nonlinear stiffness terms, the electromechanical coupling coefficient, the damping, the distance between magnets, and the load resistance on the dynamic response and the energy harvesting performance of the ABEH is numerically explored. More importantly, experiments are designed to verify the energy harvesting enhancement of the ABEH. Compared with the non-magnet energy harvester, the ABEH has much better energy harvesting performance.

## 1. Introduction

Nonlinear vibration energy harvesting techniques via various mechanisms have been widely researched because of their great application potential for powering wireless sensors and small portable devices [1,2,3,4,5,6]. Especially, piezoelectric vibration energy harvesting from base vibrations, flow-induced vibrations, human motions, has been receiving more and more attention [7,8,9,10,11,12]. In order to improve energy harvesting performance, many different kinds of linear resonance-based piezoelectric vibration energy harvesters were designed. Erturk and Inman [13] firstly derived the exact distributed parameter model for the cantilever beam-based energy harvesters with experimental verification. In order to power cardiac pacemakers, Karami and Inman [14,15,16] designed piezoelectric energy harvesters based on zigzag structures. Wickenheiser [17] presented a transfer matrix method for obtaining analytical solutions of beam-based structures with pointwise discontinuities, bends, or lumped inertias between members or the tip of the structures. For enhanced multi-directional energy harvesting, Zhou et al. [18,19] designed a flexible longitudinal zigzag energy harvester and derived an exact theoretical model which was checked by finite element method and experiments. Yang et al. [20] designed an arc-shaped piezoelectric energy harvester to improve energy harvesting efficiency. The design can also efficiently harvest energy from multi-directional vibrations.

Although the above linear energy harvesters work well when the frequency of ambient vibrations matches their natural frequencies, the energy harvesting efficiency will sharply decrease for the broadband excitations [21,22]. However, many application environments have broadband or random vibrations, which bring difficulty of energy harvesting. This challenging issue inspires new designs of high-efficiency energy harvesters based on nonlinearities [23,24,25,26,27,28,29,30]. The bistable energy harvester (BEH) whose snap-through behavior can greatly enhance energy harvesting performance is one of most well-known nonlinear energy harvesters. One enhanced energy harvesting characteristic is from the stochastic resonance of bistable systems [31,32]. Based on this, a BEH was designed and tested under random excitations, and the excellent performance was experimentally verified [33]. The detailed enhancement of the BET based on stochastic resonance was numerically and experimentally analyzed by Litak et al. [34,35]. Vocca et al. [36] digitally simulated output power of the BEH to random vibrations from non-equilibrium thermal noise up to machine vibrations and the superior performance was obtained. He and Daqaq [37] used statistical linearization, direct numerical integration of the stochastic differential equations, and the Fokker–Plank–Kolmogorov equation to reveal the influence mechanism of the potential energy function on mean output power of the BEH under white noise. The comparison of nonlinear monostable energy harvester and the BEH demonstrates that the performance of BEH is better under some random excitations [38,39].

Under harmonic excitations, the snap-through behavior induces high-energy interwell oscillation of the BEH, which greatly enhances the energy harvesting performance. This was experimentally verified by Erturk and Inman [40,41]. Stanton et al. [42] derived a complete distributed parameter model for the magnetic coupled BEH to predict its output voltage and nonlinear dynamic behavior. Under different harmonic excitations, broadband characteristics and multi-solution range can be numerically and experimentally observed [43]. Based on the harmonic balance method, the analytical solutions and corresponding stability analysis conditions of the BEH could be derived [44,45]. Other kinds of BEHs also has high-energy interwell oscillations and large-amplitude output voltage [46,47,48]. When it connects with self-powered nonlinear interface circuits, the output power of the BEH can still surpass the traditional linear ones [49,50]. More importantly, experimental tests show that the BEH has excellent performance for energy harvesting from the human body to power embedded medical devices [51,52].

In order to enhance vibration energy harvesting, this paper designs an arc-shaped piezoelectric bistable vibration energy harvester. In Section 2, a theoretical model is built. In Section 3, the influence of system parameters and excitation conditions on the dynamic response and energy harvesting performance of the ABEH is numerically explored. In Section 4, experimental verification is provided. Finally, key conclusions are addressed.

## 2. Theoretical Modeling

### 2.1. Nonlinear Magnetic Force Model

**Figure 1:**
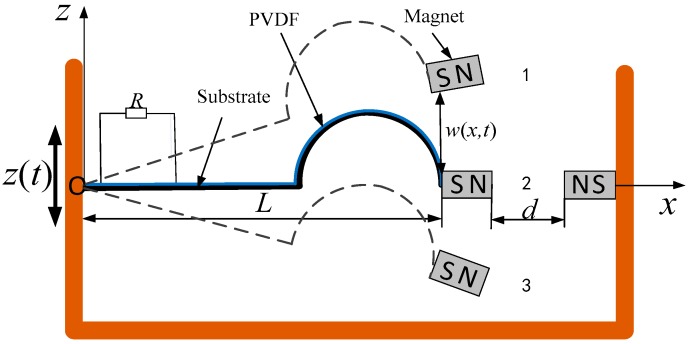
Schematic diagram of the presented ABEH.

As shown in Figure 1, the presented arc-shaped piezoelectric bistable vibration energy harvester (ABEH) consists of an arc-shaped piezoelectric cantilever beam [20], a load resistance, a tip magnet and an external magnet. The ABEH is installed in a base structure which transfers the base excitation *z*(*t*) to the harvester. Thus, the deformation direction of the beam is in the *z* direction. The flexible piezoelectric material polyvinylidene fluoride (PVDF) covers the whole beam to convert vibration energy into electric energy. The length of the horizontal part of the arc-shaped beam is *L*, and the mass of the tip magnet is *m*. w(x,t) is used to describe the vibration displacement of the ABEH.

Figure 1 shows that large transverse vibration and axial stretching vibration of the ABEH will be generated under base excitations. To improve the precision of the theoretical model, this paper fully considers the magnetic nonlinearity and the structural nonlinearity of the ABEH in the modeling process. Several assumptions should be given: The ABEH complies with the Euler Bernoulli beam theory. The shear deformation and the rotary inertia will be neglected because the thickness of the ABEH is much smaller than its length. The cross section of the ABEH keeps in a plane when it deforms and is perpendicular to plane of geometric axis.

**Figure 2:**
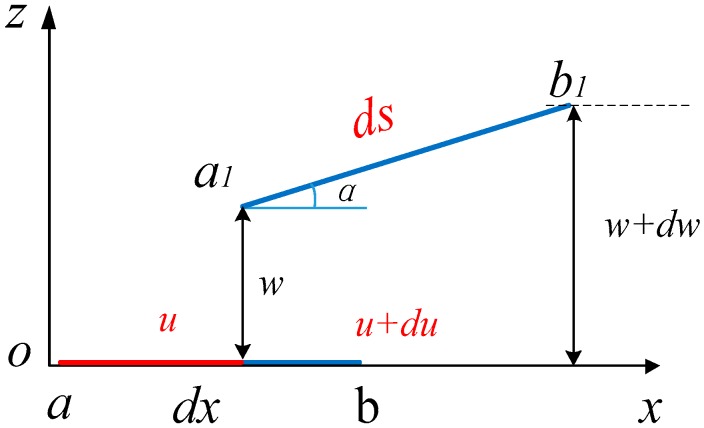
Assumed geometric relationship.

Without magnetic coupling, the piezoelectric beam is simplified for calculating its strain. Considering the axial deformation of the beam, the geometric relationship before and after deformation is shown in Figure 2 [53]. u(x,t) and w(x,t) are the vibration displacements of the piezoelectric beam along *x* axis and *z* axis, respectively. The element segment is represented by *a* and *b*, and *a*_1_ and *b*_1_ stand for the piezoelectric beam after movement. The *x* axis displacement and the *z* axis displacement caused by deformation are expressed by *u* and *w*, respectively.

The deformed section of the piezoelectric beam is ds, and rotation angle is *α*. The relation between u(x,t) and w(x,t) can be expressed as:

(1) $$ds=\sqrt{{\left(1+\frac{du}{dx}\right)}^{2}+{\left(\frac{d\omega }{dx}\right)}^{2}}\approx \sqrt{1+{\left(\frac{d\omega }{dx}\right)}^{2}}$$

The strain along with the *x* axis caused by the stretching force can be expressed as:

(2) $$\frac{ds-dx}{dx}=\sqrt{1+{\left(\frac{d\omega }{dx}\right)}^{2}}-1\approx \frac{1}{2}{\left(\frac{d\omega }{dx}\right)}^{2}$$

The bending strain in the *z* axis can be defined as:

(3) $$S_{z}=-z\frac{d\alpha }{ds}=-z\left(\frac{\partial^{2}\omega \left(x,t\right)}{\partial x^{2}}{\left(1+{\left(\frac{d\omega }{dx}\right)}^{2}\right)}^{-\frac{3}{2}}\right)\approx -z\frac{\partial^{2}\omega \left(x,t\right)}{\partial x^{2}}$$

Thus, the geometric deformation relationship of strains is:

(4) $$S_{xz}=-z\frac{\partial^{2}\omega \left(x,t\right)}{\partial x^{2}}+\frac{1}{2}{\left(\frac{\partial \omega \left(x,t\right)}{\partial x}\right)}^{2}$$

where *z* is the distance from the surface of the piezoelectric beam to the neutral layer. Then:

(5) $$z=\frac{h_{s}}{2}+h_{p}$$

Assuming that the PVDF is completely attached to the upper surface of the cantilever beam, and the electric field intensity is uniformly distributed. The electric field intensity can be represented as:

(6) $$E_{3}\left(t\right)=-\frac{v\left(t\right)}{h_{p}}$$

where v(t) denotes the output voltage, and $h_{p}$ is the thickness of the PVDF.

**Figure 3:**
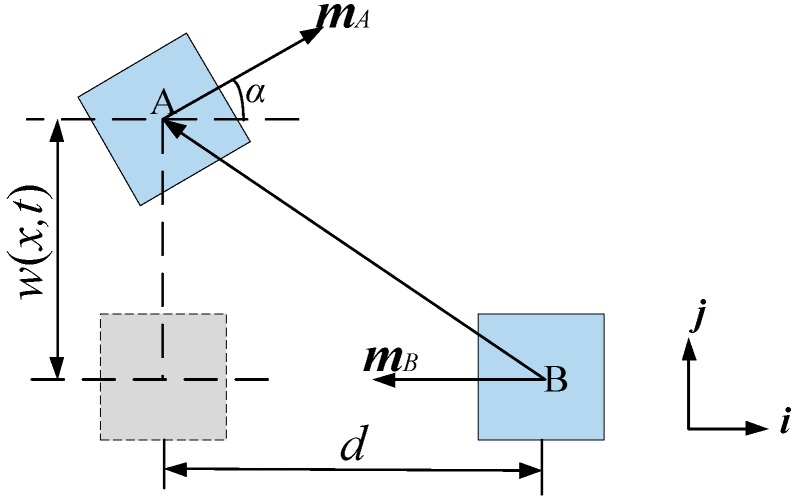
Magnetic field model.

The geometric relationship between two permanent magnets is shown in Figure 3 [54]. Considering the position vector from magnet B to magnet A, the assumed model between magnetic dipoles is used to analyze the nonlinear magnetic force.

The intensity of magnetization generated by magnet B on magnet A is:

(7) $$B_{BA}=\frac{\mu_{0}}{4\pi }\left[\frac{3\left(m_{B}\cdot r_{BA}\right)r_{BA}}{|r_{BA}{|}^{5}}-\frac{m_{B}}{|r_{BA}{|}^{3}}\right]$$

where $\mu_{0}=4\pi \times 10^{-7}\;H/m$ is the magnetic permittivity. $m_{A}\left(m_{Ax},m_{Ay},m_{Az}\right)$ and $m_{B}\left(m_{Bx},m_{By},m_{Bz}\right)$ are the magnetic dipole moment of magnets A and B, respectively. $r_{BA}(xi+yj+zk)$ is the position vector from magnet B to magnet A. The inclination angle of magnet A is α, and ω(x,t) is the vibration response amplitude. $m_{A}$, $m_{B}$ and $r_{BA}$ can be expressed as: $m_{A}=[M_{A}V_{A}\cos\alpha ,M_{A}V_{A}\sin\alpha ,0]$, $m_{B}=\left[-M_{B}V_{B},0,0\right]$, $r_{BA}=\left[-d,w,0\right]$. $M_{A}\;\mathrm{and}\;M_{B}$ are the magnetization of magnets A and B, respectively. $V_{A}\;\mathrm{and}\;V_{B}$ are the volume of magnets A and B, respectively.

Therefore, the relationship among magnet A, deflection angle *α* and amplitude ω(x,t) can be defined as:

(8) $$\tan\alpha \left(t\right)=\frac{\partial \omega \left(x,t\right)}{\partial x}$$

Thus, the magnetic potential energy is:

(9) $$U_{M_{t}}=-B_{BA}\cdot m_{A}=\frac{\mu_{0}M_{A}V_{A}M_{B}V_{B}\left(2d^{2}-3d\omega \left(x,t\right)\frac{\partial \omega \left(x,t\right)}{\partial x}-\omega {\left(x,t\right)}^{2}\right)}{4\pi \sqrt{\left({\frac{\partial \omega \left(x,t\right)}{\partial x}}^{2}+1\right)}{\left(\omega {\left(x,t\right)}^{2}+d^{2}\right)}^{5/2}}$$

### 2.2. Modal Shape for Bending Vibrations

In order to obtain the governing equations of the ABEH, we should firstly get the modal shape. The transverse vibration displacement ω(x,t) in the *z* direction of the modal shape can be expressed as [1]:

(10) $$\omega \left(x,t\right)=\overset{N}{\underset{i=1}{\sum }}\emptyset_{i}\left(x\right)r_{i}\left(t\right)$$

where $\emptyset_{i}\left(x\right)$ is the mode shape. $r_{i}\left(t\right)$ is the modal coordinates. N is the number of the mode shape.

$\emptyset_{i}\left(x\right)$ is defined as [13]:

(11) $$\emptyset_{i}\left(x\right)=A_{i}\left[\cos\frac{\lambda_{i}}{L}x-\cos h\frac{\lambda_{i}}{L}x+\zeta_{i}\left(\sin\frac{\lambda_{i}}{L}x-\sin h\frac{\lambda_{i}}{L}x\right)\right]$$

At the clamped end, the displacement and the rotation angle should be zero: $\emptyset_{i}\left(x\right)=0$ and ${\emptyset_{i}}^{\prime }\left(0\right)=0$.

Based on the boundary conditions at the free end, $\emptyset_{i}\left(x\right)$ can be simplified as [13]:

(12) $$\emptyset_{i}\left(x\right)=1-\cos\left[\frac{\left(2i-1\right)\pi x}{2L}\right]$$

The first vibration mode of the cantilever-based energy harvester was theoretically and experimentally verified to play an overwhelming role [1,13,18,19]. Therefore, this paper only considers the first vibration mode. Therefore, ω(x,t) and the modal shape can respectively be expressed as:

(13) $$\omega \left(x,t\right)=\emptyset_{1}\left(x\right)r_{1}\left(t\right)$$

(14) $$\emptyset_{1}\left(x\right)=1-\cos\left(\frac{\pi x}{2L}\right)$$

Based on Equations (9), (13) and (14), the potential energy function can be represented as:

(15) $$U_{M_{t}}=\frac{\mu_{0}M_{A}V_{A}M_{B}V_{B}}{4\pi }\times \frac{\left(2d^{2}-3d\emptyset_{1}\left(x\right){r_{1}}^{2}\left(t\right){\emptyset_{1}}^{\prime }\left(x\right)-{\emptyset_{1}}^{2}\left(x\right){r_{1}}^{2}\left(t\right)\right)}{\sqrt{\left({\emptyset_{1}}^{\prime }{\left(x\right)}^{2}{r_{1}}^{2}\left(t\right)+1\right)}{\left({\emptyset_{1}}^{2}\left(x\right){r_{1}}^{2}\left(t\right)+d^{2}\right)}^{5/2}}$$

The parameter ${r_{1}}^{2}\left(t\right)$ is set as an independent variable and the Taylor series expansion of Equation (15) is given by:

(16) $$U_{M_{t}}=\frac{\mu_{0}M_{A}V_{A}M_{B}V_{B}}{2\pi d^{3}}-\frac{1}{2}K_{1}{r_{1}}^{2}\left(t\right)+\frac{1}{4}K_{2}{r_{1}}^{4}\left(t\right)$$

The derivative of Equation (16) is:

(17) $$\delta U_{M_{t}}=-K_{1}r_{1}\left(t\right)+K_{2}{r_{1}}^{3}\left(t\right)$$

where:

(18) $$K_{1}=\frac{\mu_{0}M_{A}V_{A}M_{B}V_{B}}{4\pi }\times \frac{\left(12{\emptyset_{1}}^{2}\left(L\right)+2{\emptyset_{1}}^{\prime }{\left(L\right)}^{2}d^{2}+6\emptyset_{1}\left(L\right){\emptyset_{1}}^{\prime }\left(L\right)d\right)}{d^{5}}$$

(19) $$\begin{array}{c} K_{2}=\frac{\mu_{0}M_{A}V_{A}M_{B}V_{B}}{4\pi }\times (\frac{45{\emptyset_{1}}^{4}\left(L\right)+15{\emptyset_{1}}^{\prime }\left(L\right){\emptyset_{1}}^{3}\left(L\right)d}{d^{7}}+\cdots \\ \;\frac{12{\emptyset_{1}}^{\prime }{\left(L\right)}^{2}{\emptyset_{1}}^{2}\left(L\right)d^{2}+6{\emptyset_{1}}^{\prime }{\left(L\right)}^{3}\emptyset_{1}\left(L\right)d^{3}}{d^{7}}+\frac{3{\emptyset_{1}}^{\prime }{\left(L\right)}^{4}d^{4}}{d^{7}}) \end{array}$$

The strain $S_{1}$ and the electric field strength $E_{3}$ can be simplified as:

(20) $$S_{1}\left(x,z,t\right)=-z{\emptyset_{1}}^{\prime \prime }\left(x\right)r_{1}\left(t\right)+\frac{1}{2}{\emptyset_{1}}^{\prime }{\left(x\right)}^{2}r_{1}{\left(t\right)}^{2}$$

(21) $$E_{3}\left(t\right)=-\frac{v\left(t\right)}{h_{p}}$$

### 2.3. Complete Governing Model

Based on Hooke’s law, the stress-strain relationship of the cantilever beam is expressed as:

(22) $$T_{1}=Y_{s}S_{1}$$

where $Y_{s}$ is the Young’s modulus of the substrate layer. $T_{1}$ and $S_{1}$ are the stress and the strain components along the *x* direction, respectively.

When the harvester vibrates in the *z* direction, the second type piezoelectric equation is given by:

(23) $$T_{1}={\bar{c}}_{11}^{E}S_{1}-{\bar{e}}_{31}E_{3}$$

(24) $$D_{3}={\bar{e}}_{31}S_{1}+{\bar{\varepsilon }}_{33}^{s}E_{3}$$

where $T_{1}$ and $S_{1}$ are the stress and strain components along the *x* direction of PVDF, respectively. ${\bar{c}}_{11}^{E}$ is the Young’s modulus of PVDF. ${\bar{e}}_{31}$ is piezoelectric coupling coefficient. $E_{3}$ and $D_{3}$ are the electric field and the electric displacement vector, respectively. ${\bar{\varepsilon }}_{33}^{s}$ is the dielectric permittivity at constant stress.

When the mechanical dissipation effect is ignored, the internal electric energy is defined as:

(25) $$\delta S=\overset{t_{2}}{\underset{t_{1}}{\int }}\left(\delta L+\delta W_{nc}\right)d_{t}=0$$

where $L=T-U+W_{ie}$ is Lagrange function and $\delta L=\delta T-\delta U+\delta W_{ie}$. T, U and $W_{ie}$ are the total kinetic energy, potential energy and electric energy of the system, respectively. $W_{nc}$ is the virtual work of non-conservative mechanical force and electric charge in the system.

The total kinetic energy *T* is composed of the kinetic energy of the substrate $T_{S}$, the kinetic energy of PVDF $T_{p}$ and the kinetic energy of the permanent magnet at the free end of the ABEH $T_{Mt}$.

Thus, the kinetic energy $T_{S}$ is:

(26) $$T_{S}=\frac{1}{2}\rho_{S}\int_{V_{S}}{\left(\frac{\partial u\left(x,t\right)}{\partial t}\right)}^{2}d_{V_{S}}=\frac{1}{2}\rho_{S}A_{s}\overset{L}{\underset{0}{\int }}{\left(\dot{\omega }\left(x,t\right)+\dot{z}\left(t\right)\right)}^{2}d_{x}$$

where $\rho_{S}$ and $A_{S}$ are the density and cross-sectional areas of the substrate, respectively. u(x,t) denotes the vibration amplitude at the free end of the ABEH. $\dot{z}\left(t\right)$ is the velocity of the base excitation. ω(x,t) is the vibration amplitude of the ABEH in the *z* direction.

The kinetic energy $T_{p}$ is:

(27) $$T_{p}=\frac{1}{2}\rho_{p}\int_{V_{p}}{\left(\frac{\partial u\left(x,t\right)}{\partial t}\right)}^{2}d_{V_{p}}=\frac{1}{2}\rho_{p}A_{p}\overset{L}{\underset{0}{\int }}{\left(\dot{\omega }\left(x,t\right)+\dot{z}\left(t\right)\right)}^{2}d_{x}$$

where $\rho_{p}$ and $A_{p}$ are the density and cross-sectional areas of PVDF, respectively.

The kinetic energy $T_{Mt}$ is:

(28) $$T_{M_{t}}=\frac{1}{2}M_{t}{\left(\dot{\omega }\left(x,t\right){/}_{x=L}+\dot{z}\left(t\right)\right)}^{2}$$

where $M_{t}$ is the mass of magnet A.

Consequently, the total kinetic energy T is given by

(29) $$\begin{array}{cc} T=T_{S}+T_{p}+T_{M_{t}} & =\frac{1}{2}\rho_{S}A_{s}\overset{L}{\underset{0}{\int }}{\left(\dot{\omega }\left(x,t\right)+\dot{z}\left(t\right)\right)}^{2}d_{x}+\cdots \\ & \;\frac{1}{2}\rho_{p}A_{p}\overset{L}{\underset{0}{\int }}{\left(\dot{\omega }\left(x,t\right)+\dot{z}\left(t\right)\right)}^{2}d_{x}+\frac{1}{2}M_{t}{\left(\dot{\omega }\left(x,t\right){/}_{x=L}+\dot{z}\left(t\right)\right)}^{2} \end{array}$$

Based on Equation (23), the potential energy of substrate is represented as:

(30) $$U_{s}=\frac{1}{2}\underset{V_{S}}{\int }S_{1}T_{1}d_{V_{S}}=\frac{1}{2}Y_{s}A_{s}\int_{0}^{L}{\left(-z\frac{\partial^{2}\omega \left(x,t\right)}{\partial x^{2}}+\frac{1}{2}{\left(\frac{\partial \omega \left(x,t\right)}{\partial x}\right)}^{2}\right)}^{2}d_{x}$$

where $Y_{s}$, $T_{1}$ and $S_{1}$ are the Young’s modulus, the stress and the strain of the substrate, respectively.

The potential energy of PVDF can be given by the combination of Equations (20), (23) and (24):

(31) $$\begin{array}{cc} U_{p}=\frac{1}{2}\underset{V_{p}}{\int }S_{1}T_{1}d_{V_{p}}= & \frac{1}{2}\underset{V_{p}}{\int }S_{1}\left({\bar{c}}_{11}^{E}S_{1}-{\bar{e}}_{31}E_{3}\right)d_{V_{p}}=\cdots \\ & \frac{1}{2}{\bar{c}}_{11}^{E}A_{p}\int_{0}^{L}{\left(-z\frac{\partial^{2}\omega \left(x,t\right)}{\partial x^{2}}+\frac{1}{2}{\left(\frac{\partial \omega \left(x,t\right)}{\partial x}\right)}^{2}\right)}^{2}d_{x}+\cdots \\ & \;\frac{1}{2}{\bar{e}}_{31}A_{p}\frac{v\left(t\right)}{h_{p}}\int_{0}^{L}\left(-z\frac{\partial^{2}\omega \left(x,t\right)}{\partial x^{2}}+\frac{1}{2}{\left(\frac{\partial \omega \left(x,t\right)}{\partial x}\right)}^{2}\right)d_{x} \end{array}$$

The total potential energy of the ABEH is:

(32) $$\begin{array}{cc} U=U_{s}+U_{p}+U_{M_{t}}= & \frac{1}{2}Y_{s}A_{s}\int_{0}^{L}{\left(-z\frac{\partial^{2}\omega \left(x,t\right)}{\partial x^{2}}+\frac{1}{2}{\left(\frac{\partial \omega \left(x,t\right)}{\partial x}\right)}^{2}\right)}^{2}d_{x}+\cdots \\ & \frac{1}{2}{\bar{c}}_{11}^{E}A_{p}\int_{0}^{L}{\left(-z\frac{\partial^{2}\omega \left(x,t\right)}{\partial x^{2}}+\frac{1}{2}{\left(\frac{\partial \omega \left(x,t\right)}{\partial x}\right)}^{2}\right)}^{2}d_{x}+\cdots \\ & \;\frac{1}{2}{\bar{e}}_{31}A_{p}\frac{v\left(t\right)}{h_{p}}\int_{0}^{L}\left(-z\frac{\partial^{2}\omega \left(x,t\right)}{\partial x^{2}}+\frac{1}{2}{\left(\frac{\partial \omega \left(x,t\right)}{\partial x}\right)}^{2}\right)d_{x}+U_{M_{t}} \end{array}$$

Combined Equations (21), (23) and (24), the electric energy generated by PVDF is:

(33) $$\begin{array}{c} W_{ie}=\frac{1}{2}\underset{v_{p}}{\int }E_{3}D_{3}d_{v_{p}}=\frac{1}{2}A_{p}\int_{0}^{L}E_{3}\left({\bar{e}}_{31}S_{1}+{\bar{\varepsilon }}_{33}^{s}E_{3}\right)d_{x}=\cdots \\ \;-\frac{1}{2}{\bar{e}}_{31}A_{p}\frac{v\left(t\right)}{h_{p}}\int_{0}^{L}\left(-z\frac{\partial^{2}\omega \left(x,t\right)}{\partial x^{2}}+\frac{1}{2}{\left(\frac{\partial \omega \left(x,t\right)}{\partial x}\right)}^{2}\right)d_{x}+\frac{1}{2}c_{p}v^{2}\left(t\right) \end{array}$$

where $E_{3}$ is the electric field intensity of PVDF in the *z* direction. $D_{3}$ is the electric displacement of PVDF. The internal capacitance of PVDF $c_{p}$ is given by:

(34) $$c_{p}={\bar{\varepsilon }}_{33}^{s}\frac{A_{p}}{h_{p}}$$

In this paper, a linear damping with damping coefficient *c* is assumed [55]. The virtual work of non-conservative mechanical force is:

(35) $$w_{c}=\int_{0}^{L}\left(-c\omega \left(x,t\right)\frac{\partial \omega \left(x,t\right)}{\partial x}\right)d_{x}$$

The virtual work of the electric charge is mainly caused by the external load resistance of the ABEH, which can be expressed as:

(36) $$W_{R}=Q\left(t\right)v\left(t\right)$$

Thus, the virtual work of non-conservative mechanical force and electric charge is:

(37) $$W_{nc}=\int_{0}^{L}\left(-c\omega \left(x,t\right)\frac{\partial \omega \left(x,t\right)}{\partial t}\right)d_{x}+Q\left(t\right)v\left(t\right)$$

where v(t) and Q(t) are respectively the voltage and the quantity of electric charge generated by PVDF.

Combined Equations (13), (14), (29), (32), Equations (33) and (37) can be simplified as:

(38) $$\begin{array}{c} T=\frac{1}{2}\left(\rho_{S}A_{s}+\rho_{p}A_{p}\right)\int_{0}^{L}\left(\emptyset_{1}{\left(x\right)}^{2}\dot{r_{1}}{\left(t\right)}^{2}+2\emptyset_{1}\left(x\right)\dot{r_{1}}\left(t\right)\dot{z}\left(t\right)+\dot{z}{\left(t\right)}^{2}\right)d_{x}+\cdots \\ \;\frac{1}{2}M\left(\emptyset_{1}{\left(x\right)}^{2}\dot{r_{1}}{\left(t\right)}^{2}+2\emptyset_{1}\left(x\right)\dot{r_{1}}\left(t\right)\dot{z}\left(t\right)+\dot{z}{\left(t\right)}^{2}\right) \end{array}$$

(39) $$\begin{array}{c} U=\frac{1}{2}\left(Y_{s}A_{s}+{\bar{c}}_{11}^{E}A_{p}\right)\int_{0}^{L}\left(z^{2}{\emptyset_{1}}^{\prime \prime }{\left(x\right)}^{2}r_{1}{\left(t\right)}^{2}-z{\emptyset_{1}}^{\prime \prime }\left(x\right){\emptyset_{1}}^{\prime }{\left(x\right)}^{2}r_{1}{\left(t\right)}^{3}+\frac{1}{4}{\emptyset_{1}}^{\prime }{\left(x\right)}^{4}r_{1}{\left(t\right)}^{4}\right)d_{x}+\cdots \\ \;\frac{1}{2}{\bar{e}}_{31}A_{p}\frac{v\left(t\right)}{h_{p}}\int_{0}^{L}\left(-z{\emptyset_{1}}^{\prime \prime }\left(x\right)r_{1}\left(t\right)+\frac{1}{2}{\emptyset_{1}}^{\prime }{\left(x\right)}^{2}r_{1}{\left(t\right)}^{2}\right)d_{x}+U_{M_{t}} \end{array}$$

(40) $$W_{ie}=-\frac{1}{2}{\bar{e}}_{31}A_{p}\frac{v\left(t\right)}{h_{p}}\int_{0}^{L}\left(-z{\emptyset_{1}}^{\prime \prime }\left(x\right)r_{1}\left(t\right)+\frac{1}{2}{\emptyset_{1}}^{\prime }{\left(x\right)}^{2}r_{1}{\left(t\right)}^{2}\right)d_{x}+\frac{1}{2}c_{p}v^{2}\left(t\right)$$

(41) $$W_{nc}=\int_{0}^{L}\left(-c\emptyset_{1}{\left(x\right)}^{2}r_{1}\left(t\right)\dot{r_{1}}\left(t\right)\right)d_{x}+Q\left(t\right)v\left(t\right)$$

Therefore, the Lagrange function can be expressed as:

(42) $$\begin{array}{l} L=T-U+W_{ie}=\left(\frac{1}{2}\left(\rho_{S}A_{s}+\rho_{p}A_{p}\right)\int_{0}^{L}\emptyset_{1}{\left(x\right)}^{2}d_{x}+\frac{1}{2}M\emptyset_{1}{\left(x\right)}^{2}\right)\dot{r_{1}}{\left(t\right)}^{2}-\cdots \\ \;\frac{1}{2}\left(\left(Y_{s}A_{s}+{\bar{c}}_{11}^{E}A_{p}\right)\int_{0}^{L}z^{2}{\emptyset_{1}}^{\prime \prime }{\left(x\right)}^{2}d_{x}\right)r_{1}{\left(t\right)}^{2}-\frac{1}{2}{\bar{e}}_{31}A_{p}\frac{v\left(t\right)}{h_{p}}\int_{0}^{L}{\emptyset_{1}}^{\prime }{\left(x\right)}^{2}d_{x}r_{1}{\left(t\right)}^{2}+\cdots \\ \;\frac{1}{2}\left(Y_{s}A_{s}+{\bar{c}}_{11}^{E}A_{p}\right)\int_{0}^{L}\left(z{\emptyset_{1}}^{\prime \prime }\left(x\right){\emptyset_{1}}^{\prime }{\left(x\right)}^{2}\right)d_{x}r_{1}{\left(t\right)}^{3}-\frac{1}{8}\left(Y_{s}A_{s}+{\bar{c}}_{11}^{E}A_{p}\right)\int_{0}^{L}{\emptyset_{1}}^{\prime }{\left(x\right)}^{4}d_{x}r_{1}{\left(t\right)}^{4}+\cdots \\ \;{\bar{e}}_{31}A_{p}z\frac{1}{h_{p}}\int_{0}^{L}{\emptyset_{1}}^{\prime \prime }\left(x\right)d_{x}v\left(t\right)r_{1}\left(t\right)+\left(\left(\rho_{S}A_{s}+\rho_{p}A_{p}\right)\int_{0}^{L}\emptyset_{1}\left(x\right)dx+M\emptyset_{1}\left(x\right)\right)\dot{r_{1}}\left(t\right)\dot{z}\left(t\right)+\cdots \\ \;\left(\frac{1}{2}\left(\rho_{S}A_{s}+\rho_{p}A_{p}\right)L+\frac{1}{2}M\right)\dot{z}{\left(t\right)}^{2}+\frac{1}{2}c_{p}v^{2}\left(t\right)-\left(\frac{\mu_{0}M_{A}V_{A}M_{B}V_{B}}{2\pi d^{3}}-\frac{1}{2}K_{1}{r_{1}}^{2}\left(t\right)+\frac{1}{4}K_{2}{r_{1}}^{4}\left(t\right)\right) \end{array}$$

Equation (42) is simplified as

(43) $$\begin{array}{l} L=\frac{1}{2}M\dot{r_{1}}{\left(t\right)}^{2}-\frac{1}{2}Kr_{1}{\left(t\right)}^{2}-\frac{1}{2}\vartheta_{1}v\left(t\right)r_{1}{\left(t\right)}^{2}+\frac{1}{3}N_{1}r_{1}{\left(t\right)}^{3}-\frac{1}{4}N_{2}r_{1}{\left(t\right)}^{4}+\vartheta_{2}v\left(t\right)r_{1}\left(t\right)+\cdots \\ \;\beta \dot{r_{1}}\left(t\right)\dot{z}\left(t\right)+\frac{1}{2}\Gamma \dot{z}{\left(t\right)}^{2}+\frac{1}{2}c_{p}v^{2}\left(t\right)-\left(\frac{\mu_{0}M_{A}V_{A}M_{B}V_{B}}{2\pi d^{3}}-\frac{1}{2}K_{1}{r_{1}}^{2}\left(t\right)+\frac{1}{4}K_{2}{r_{1}}^{4}\left(t\right)\right) \end{array}$$

The Lagrangian electromechanical equation based on the extended Hamiltonian principle is:

(44) $$\frac{d}{dt}\left(\frac{\partial T}{\partial \dot{r_{1}}}\right)-\frac{\partial T}{\partial r_{1}}+\frac{\partial U}{\partial r_{1}}-\frac{\partial W_{ie}}{\partial r_{1}}=F\left(t\right)$$

(45) $$\frac{d}{dt}\left(\frac{\partial T}{\partial \dot{v}}\right)-\frac{\partial T}{\partial v}+\frac{\partial U}{\partial v}-\frac{\partial W_{ie}}{\partial v}=Q\left(t\right)$$

The simplified form is:

(46) $$\frac{d}{dt}\left(\frac{\partial T}{\partial \dot{r_{1}}}\right)-\frac{\partial L}{\partial r_{1}}=F\left(t\right)$$

(47) $$\frac{d}{dt}\left(\frac{\partial T}{\partial \dot{v}}\right)-\frac{\partial L}{\partial v}=Q\left(t\right)$$

where F(t) is the function of non-conservative mechanical forces. Q(t) is the quantity of electric charge generated by PVDF. According Equation (35), $F\left(t\right)=-\int_{0}^{L}c\emptyset_{1}{\left(x\right)}^{2}d_{x}\dot{r_{1}}\left(t\right)$. The generalized current $\dot{Q}\left(t\right)=-v\left(t\right)/R$ is given by Kirchhoff’s law when the impedance resistance of PVDF is assumed to be R.

Based on Equations (43), (46) and (47), the electromechanical coupled equation of the ABEH can be expressed as:

(48) $$\begin{array}{l} M\ddot{r_{1}}\left(t\right)+C\dot{r_{1}}\left(t\right)+Kr_{1}\left(t\right)+\vartheta_{1}v\left(t\right)r_{1}\left(t\right)-N_{1}r_{1}{\left(t\right)}^{2}+N_{2}r_{1}{\left(t\right)}^{3}-\vartheta_{2}v\left(t\right)-\cdots \\ \;K_{1}r_{1}\left(t\right)+K_{2}r_{1}{\left(t\right)}^{3}=-\beta \ddot{z}\left(t\right) \end{array}$$

(49) $$-\vartheta_{1}\dot{r_{1}}\left(t\right)r_{1}\left(t\right)+\vartheta_{2}\dot{r_{1}}\left(t\right)+c_{p}\dot{v}\left(t\right)-v\left(t\right)/R=0$$

where M, C and K are the modal mass, the modal damping and the modal stiffness of the ABEH, respectively. They are represented as:

(50) $$M=\left(\rho_{S}A_{s}+\rho_{p}A_{p}\right)\int_{0}^{L}\emptyset_{1}{\left(x\right)}^{2}dx+M_{t}\emptyset_{1}{\left(x\right)}^{2}$$

(51) $$C=\int_{0}^{L}c\emptyset_{1}{\left(x\right)}^{2}d_{x}$$

(52) $$K=\left(Y_{s}A_{s}+{\bar{c}}_{11}^{E}A_{p}\right)\int_{0}^{L}z^{2}{\emptyset_{1}}^{\prime \prime }{\left(x\right)}^{2}d_{x}$$

$\vartheta_{1}$, $N_{1}$ and $N_{2}$ are the electromechanical coupling term, the quadratic nonlinear term and the cubic nonlinear term coefficient, respectively.

(53) $$\vartheta_{1}={\bar{e}}_{31}A_{p}\frac{1}{h_{p}}\int_{0}^{L}{\emptyset_{1}}^{\prime }{\left(x\right)}^{2}d_{x}$$

(54) $$N_{1}=\frac{3}{2}\left(Y_{s}A_{s}+{\bar{c}}_{11}^{E}A_{p}\right)\int_{0}^{L}z{\emptyset_{1}}^{\prime \prime }\left(x\right){\emptyset_{1}}^{\prime }{\left(x\right)}^{2}d_{x}$$

(55) $$N_{2}=\frac{1}{2}\left(Y_{s}A_{s}+{\bar{c}}_{11}^{E}A_{p}\right)\int_{0}^{L}{\emptyset_{1}}^{\prime }{\left(x\right)}^{4}d_{x}$$

$\vartheta_{2}$, β are respectively the electromechanical coupling coefficient and the fundamental excitation coefficient

(56) $$\vartheta_{2}={\bar{e}}_{31}A_{p}z\frac{1}{h_{p}}\int_{0}^{L}{\emptyset_{1}}^{\prime \prime }\left(x\right)d_{x}$$

(57) $$\beta =\left(\rho_{S}A_{s}+\rho_{p}A_{p}\right)\int_{0}^{L}\emptyset_{1}\left(x\right)dx+M_{t}\emptyset_{1}\left(x\right)$$

Assuming $\omega_{1}=\sqrt{\frac{K}{M}}$, $\zeta_{1}=\frac{C}{2M\omega_{1}}$, the governing equations of the ABEH can be defined, as follows:

(58) $$\begin{array}{l} \ddot{r_{1}}\left(t\right)+2\zeta_{1}\omega_{1}\dot{r_{1}}\left(t\right)+{\omega_{1}}^{2}r_{1}\left(t\right)+\frac{\vartheta_{1}}{M}v\left(t\right)r_{1}\left(t\right)-\frac{N_{1}}{M}r_{1}{\left(t\right)}^{2}+\frac{N_{2}}{M}r_{1}{\left(t\right)}^{3}-\frac{\vartheta_{2}}{M}v\left(t\right)-\frac{K_{1}}{M}r_{1}\left(t\right)+\cdots \\ \;\frac{K_{2}}{M}r_{1}{\left(t\right)}^{3}=-\frac{\beta }{M}\ddot{z}\left(t\right) \end{array}$$

(59) $$-\vartheta_{1}\dot{r_{1}}\left(t\right)r_{1}\left(t\right)+\vartheta_{2}\dot{r_{1}}\left(t\right)+c_{p}\dot{v}\left(t\right)-\frac{v\left(t\right)}{R}=0$$

In order to nondimensionalize Equations (58) and (59), the variables are standardized as:

$$r_{1}\left(t\right)=Lx\left(\tau \right),t=\frac{\tau }{\omega_{1}},v\left(t\right)=eu\left(\tau \right),e=\frac{L\vartheta_{2}}{c_{p}}$$

where L, e are standardization coefficients. Their units are meter (m) and volt (V), respectively. τ is the standardization time. Then, the governing equations become:

(60) $$\begin{array}{l} \ddot{x}\left(\tau \right)+2\zeta_{1}\dot{x}\left(\tau \right)+x\left(\tau \right)+\frac{\vartheta_{1}\vartheta_{2}L}{Kc_{p}}u\left(\tau \right)x\left(\tau \right)-\frac{N_{1}L}{K}x{\left(\tau \right)}^{2}+\frac{N_{2}L^{2}}{K}x{\left(\tau \right)}^{3}-\frac{{\vartheta_{2}}^{2}}{Kc_{p}}u\left(\tau \right)-\frac{K_{1}}{K}x\left(\tau \right)+\cdots \\ \;\frac{K_{2}L^{2}}{K}x{\left(\tau \right)}^{3}=-\frac{\beta }{KL}\ddot{z}\left(t\right) \end{array}$$

(61) $$\dot{u}\left(\tau \right)-\frac{1}{R\omega_{1}c_{p}}u\left(\tau \right)+\dot{x}\left(\tau \right)-\frac{\vartheta_{1}L}{\vartheta_{2}}\dot{x}\left(\tau \right)x\left(\tau \right)=0$$

To simplify the equations, we present the following transformations:

$$\theta_{1}=\frac{\vartheta_{1}\vartheta_{2}L}{Kc_{p}},\eta_{1}=\frac{N_{1}L}{K},\eta_{2}=\frac{N_{2}L^{2}}{K},\theta_{2}=\frac{{\vartheta_{2}}^{2}}{Kc_{p}},\kappa_{1}=\frac{K_{1}}{K},\kappa_{2}=\frac{K_{2}L^{2}}{K},f=-\frac{\beta }{KL},\Omega =\frac{\omega }{\omega_{1}},\alpha =\frac{1}{R\omega_{1}c_{p}},\Theta =\frac{\vartheta_{1}L}{\vartheta_{2}}.$$

The dimensionless governing equations of the ABEH are:

(62) $$\ddot{x}\left(\tau \right)+2\zeta_{1}\dot{x}\left(\tau \right)+x\left(\tau \right)+\theta_{1}u\left(\tau \right)x\left(\tau \right)-\eta_{1}x{\left(\tau \right)}^{2}+\eta_{2}x{\left(\tau \right)}^{3}-\theta_{2}u\left(\tau \right)-\kappa_{1}x\left(\tau \right)+\kappa_{2}x{\left(\tau \right)}^{3}=f\ddot{z}\left(t\right)$$

(63) $$\dot{u}\left(\tau \right)-\alpha u\left(\tau \right)+\dot{x}\left(\tau \right)-\Theta \dot{x}\left(\tau \right)x\left(\tau \right)=0$$

where x is the dimensionless displacement. $\zeta_{1}$ is the dimensionless linear damping. $\eta_{1},\eta_{2},\kappa_{2}$ are the dimensionless nonlinear stiffness coefficients. $\theta_{2}$ and $\theta_{1}$ are the dimensionless linear electromechanical coupling coefficient and the dimensionless nonlinear electromechanical coupling coefficient, respectively. $\kappa_{1}$ is the dimensionless linear stiffness coefficient. Θ is the dimensionless nonlinear damping coefficients.

## 3. Influence Mechanism

### 3.1. Influence of the Nonlinear Stiffness Terms

**Figure 4:**
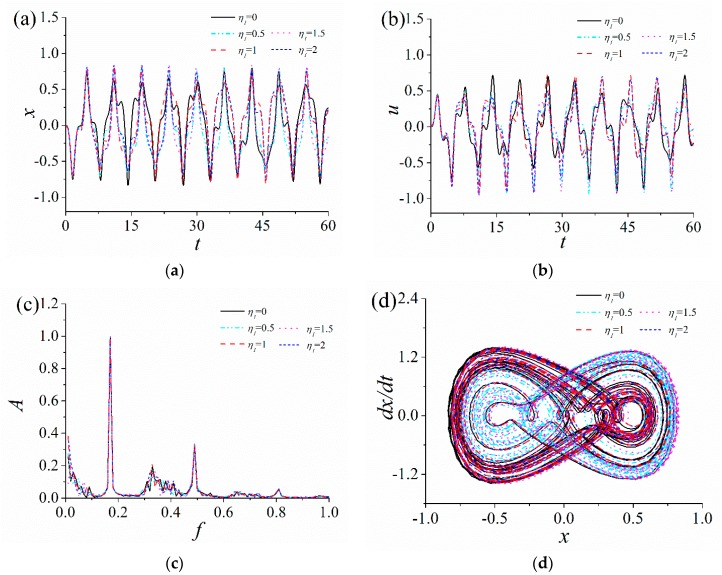
Response of the ABEH with different quadratic nonlinear stiffness coefficients $\eta_{1}$: (**a**) Displacement; (**b**) output voltage; (**c**) frequency spectrum; (**d**) phase trajectory.

**Figure 5:**
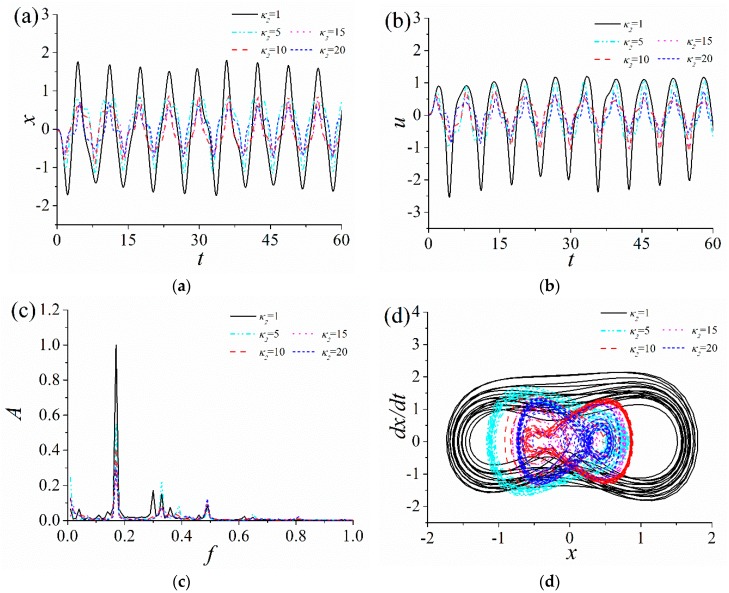
Response of the ABEH with different cubic nonlinear stiffness coefficients $\kappa_{2}$: (**a**) Displacement; (**b**) output voltage; (**c**) frequency spectrum; (**d**) phase trajectory.

In order to analyze the influence of the quadratic nonlinear stiffness coefficient $\eta_{1}$ and the cubic nonlinear stiffness coefficient $\kappa_{2}$ on the response characteristics of the ABEH, the time-domain response displacement and the output voltage, frequency spectrum and phase trajectory of the ABEH with different $\eta_{1}$ and $\kappa_{2}$ are numerically obtained, as shown in Figure 4 and Figure 5. 

It is noted that sine wave excitations are used to stimulate the ABEH in both simulations and experiments. It can be found that, the quadratic nonlinear stiffness coefficient $\eta_{1}$ has less effect on the response characteristics of the ABEH, when the multiple nonlinear stiffness term coefficients (the quadratic nonlinear stiffness coefficient $\eta_{1}$ and cubic nonlinear stiffness coefficient $\kappa_{2}$) appear simultaneously. There are a variety of harmonics in the response of the ABEH, when the cubic nonlinear stiffness coefficient $\kappa_{2}$ increases from 1. As $\kappa_{2}$ increases, the amplitudes of both displacement and output voltage decrease gradually. This indicates that the nonlinear stiffness coefficient and response of the ABEH are mutually coupled. In addition, as $\kappa_{2}$ gradually increases from 1, the response of the ABEH firstly changes from a large-amplitude interwell oscillation to a small-amplitude oscillation. This is a common nonlinear phenomenon [56,57,58,59]. It can be concluded that the cubic nonlinear stiffness coefficient $\kappa_{2}$ plays a key role in the dynamic response and the energy harvesting performance of the ABEH.

### 3.2. Influence of the Electromechanical Coupling Coefficient

**Figure 6:**
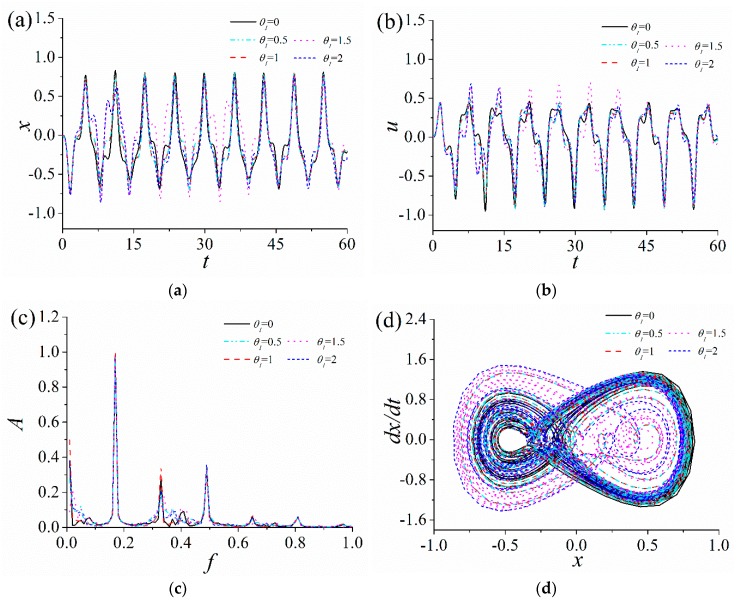
Response of the ABEH with different nonlinear electromechanical coupling coefficients $\theta_{1}$: (**a**) Displacement; (**b**) output voltage; (**c**) frequency spectrum; (**d**) phase trajectory.

**Figure 7:**
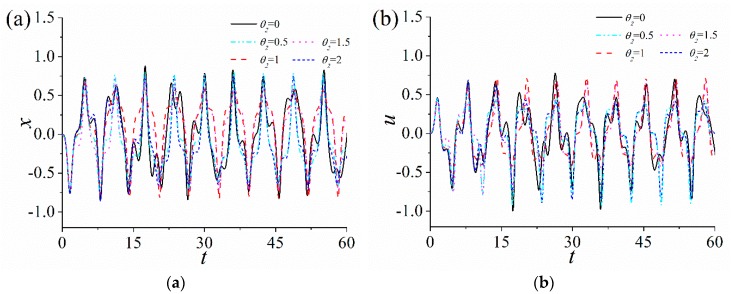

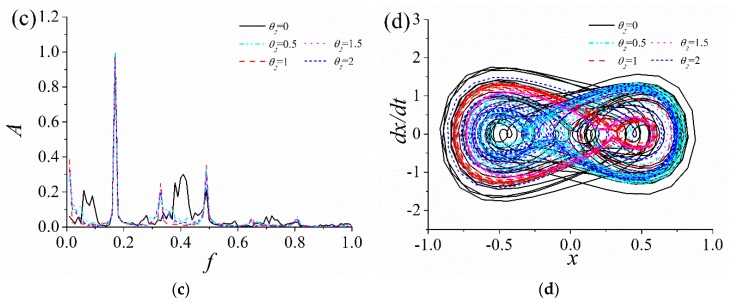
Response of the ABEH with different linear electromechanical coupling coefficients $\theta_{2}$: (**a**) Displacement; (**b**) output voltage; (**c**) frequency spectrum; (**d**) phase trajectory.

The influence of nonlinear electromechanical coupling coefficient $\theta_{1}$ and linear electromechanical coupling coefficient $\theta_{2}$ on the response characteristics of the ABEH is analyzed. The time-domain response displacement and output voltage, frequency spectrum and phase trajectory of the ABEH with different $\theta_{1}$ and $\theta_{2}$ are shown in Figure 6 and Figure 7. It is found that, $\theta_{1}$ and $\theta_{2}$ has a small effect on the response displacement and output voltage of the ABEH. As $\theta_{2}$ increases from 0, the peak value of the output voltage of the ABEH slightly increases. As $\theta_{1}$ increases, phase trajectory of the ABEH changes.

### 3.3. Influence of the Damping

**Figure 8:**
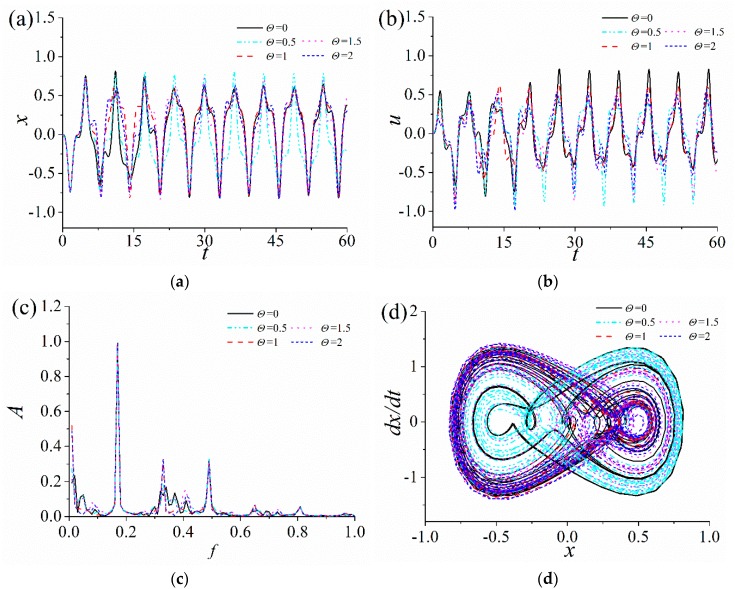
Response of the ABEH with different nonlinear damping coefficients Θ: (**a**) Displacement; (**b**) output voltage; (**c**) frequency spectrum; (**d**) phase trajectory.

In order to analyze the influence of nonlinear damping coefficient Θ on the response of the ABEH, the corresponding results are shown in Figure 8. 

It can be seen that, the output voltage of the ABEH decreases along with the increase of Θ. The reason is that the increase of Θ damps the vibration amplitude of the ABEH and more mechanical energy will be converted into heat energy. This will bring a negative influence on the energy harvesting performance of the ABEH. 

### 3.4. Influence of the Relative Positions of Magnets

**Figure 9:**
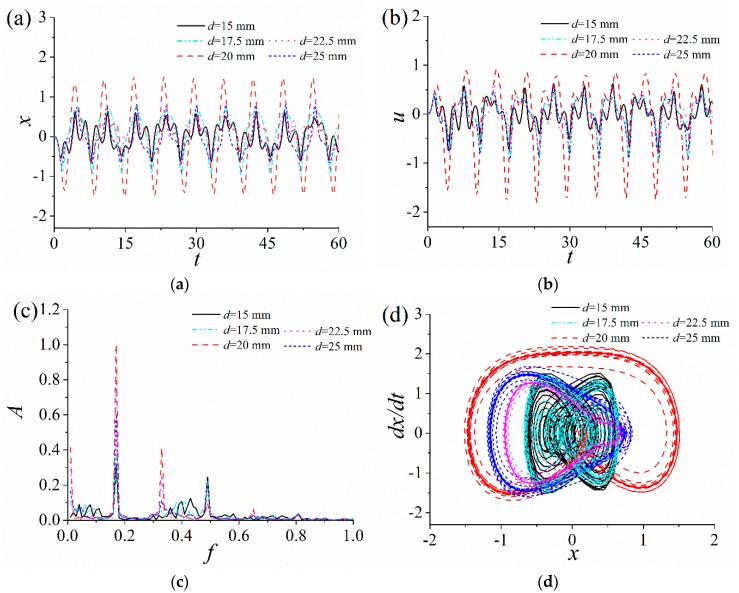
Response of the ABEH with different magnetic distance *d*: (**a**) Displacement; (**b**) output voltage; (**c**) frequency spectrum; (**d**) phase trajectory.

Above numerical results show that the relative positions of magnet play a key role in the nonlinear response characteristics of the ABEH. Therefore, in order to improve the output power, it is necessary to select a reasonable distance *d*. It is assumed that the excitation frequency Ω = 1 and amplitude *A* = 2. In addition, 15, 17.5, 20, 22.5 and 25 mm are five selected values of *d*. As the results shown in Figure 9, the displacement amplitude and the output voltage of the ABEH for *d* = 20 mm, are significantly larger than others. This means that there is an optimal *d* which make the ABEH have best output voltage and output power.

### 3.5. Influence of the Load Resistance

**Figure 10:**
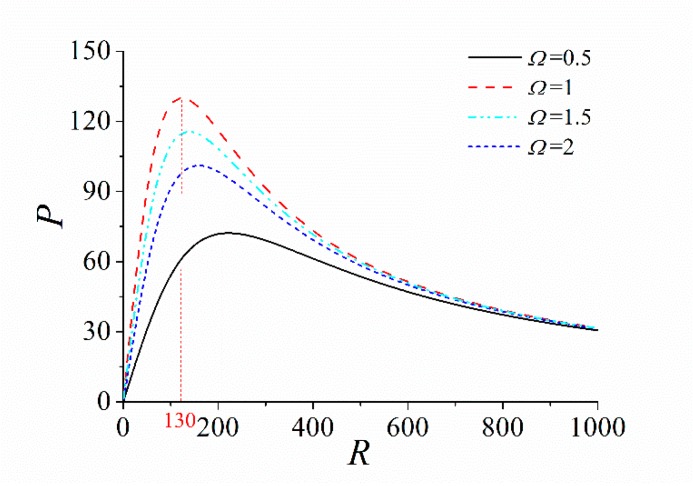
Curve of output power with load resistance with different excitation frequencies.

**Figure 11:**
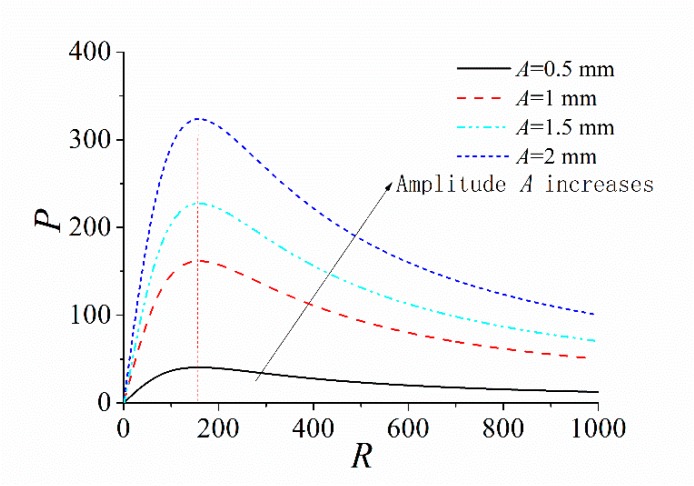
Curve of output power with load resistance with different excitation amplitudes.

It is well known that the external load resistance must match the impedance of the energy harvester to obtain the maximum output power. However, the impedance of the ABEH is not constant, which is related to the frequency and amplitude of the excitation [13]. Therefore, the numerical calculation method can be used to obtain the variation curve of the output power with load resistance for different excitation conditions. The results are shown in Figure 10 and Figure 11. It is found that the optimal load resistance changes along with the change of the excitation frequency, while the excitation amplitude has a very small influence on the optimal load resistance. Therefore, we should pay more attention on the excitation frequency when the ABEH is designed.

## 4. Experimental Verification

### Experimental Setup

**Figure 12:**
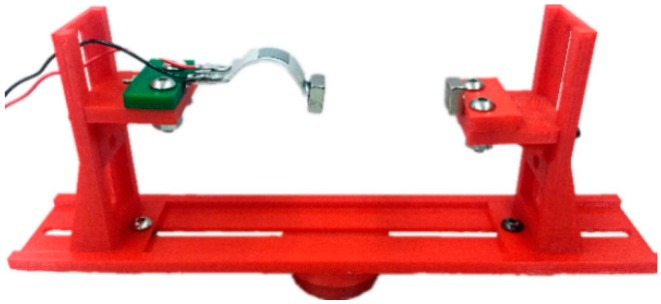
Experimental prototype of the ABEH.

**Figure 13:**
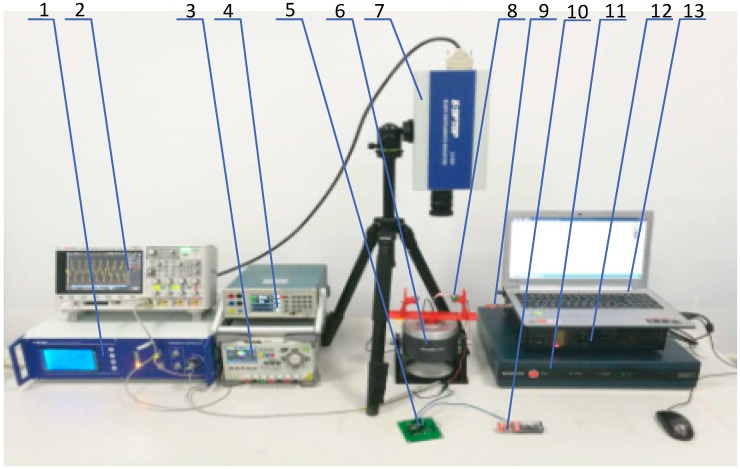
Experimental setup: 1- Laser controller; 2- Digital storage scope; 3- Programmable DC power supply; 4- Power analyzer; 5- Load resistance; 6- Vibration exciter; 7- Laser vibrometer; 8- The ABEH; 9- PC (AP end) 10- Wireless sensor module (ED end); 11- Multi-channel vibration controller; 12- Power amplifier; 13- Computer.

In order to further verify the design, a prototype of the ABEH is fabricated and shown in Figure 12. The distance *d* between the tip magnet and the external magnet can be adjusted, thus, the nonlinear characteristics of the ABEH can be changed. The ABEH is fixed on a rigid plastics support frame. Figure 13 shows the whole experimental setup. In detail, there are an ABEH, a laser doppler vibrometer, a laser controller, an acceleration sensor, a vibration exciter, a power amplifier, a vibrator control box and a computer.

**Figure 14:**
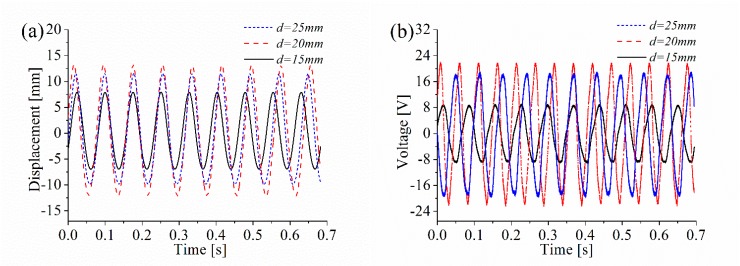

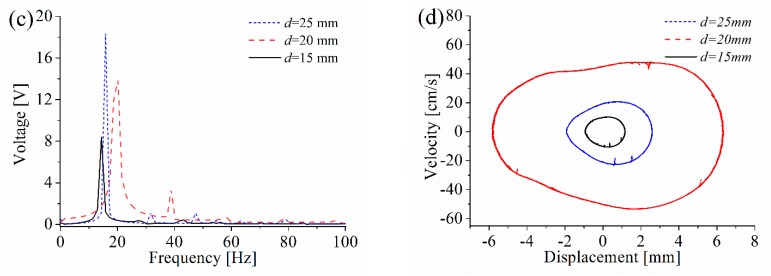
Response of the ABEH with different magnetic distance *d*: (**a**) Response displacement; (**b**) output voltage; (**c**) frequency spectrum; (**d**) phase trajectory.

**Figure 15:**
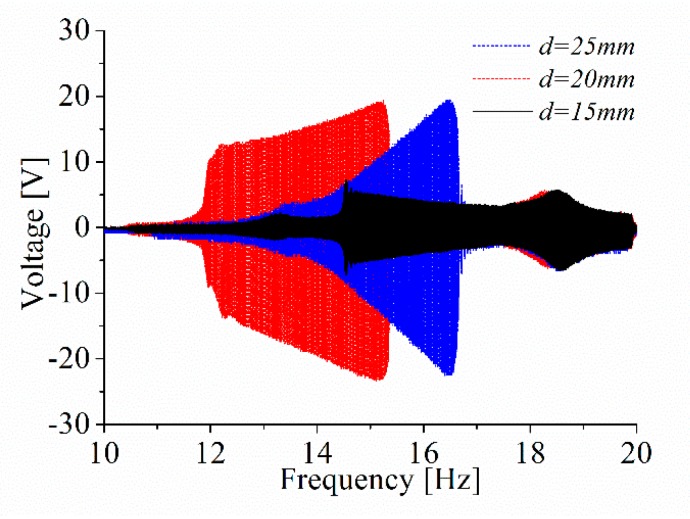
The output voltage of ABEH with different *d*.

**Figure 16:**
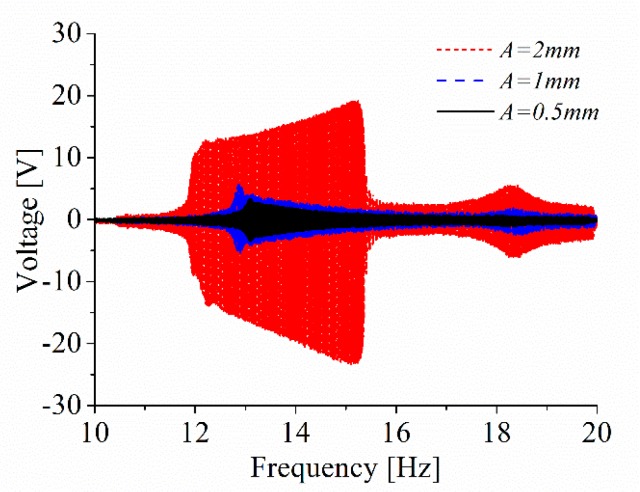
The output voltage of ABEH under different excitation amplitudes.

Firstly, the influence of the distance *d* on the energy harvesting performance of the ABEH is investigated. It can improve the energy capture efficiency and effective working frequency band of the system. For the experiments under the constant frequency excitation, it is set as 14 Hz and the excitation amplitude is set as *A* = 2 mm. In addition, the frequency-swept experiments are also performed to obtain the displacement amplitude over a wide frequency range. In accordance with numerical simulation, 15, 20 and 25 mm are three selected values of *d*. Time-domain response displacement and output voltage, displacement amplitude versus excitation frequency, and phase trajectory are shown in Figure 14. It is found that, the ABEH with *d* = 20 mm has a much wider effective frequency range where the displacement amplitude is large, compared with the cases of *d* = 15 mm and *d* = 25 mm. For the piezoelectric energy harvesting, the large displacement amplitude means the large output voltage, which can be also verified by Figure 14a,b,d. Therefore, the ABEH with *d* = 20 mm has the best energy harvesting performance among the three cases. This is further verified by Figure 15. In addition, the experimental results in Figure 15 and Figure 16 and Figure 18 are obtained from sine wave sweep excitations, which are produced by the vibration exciter. More importantly, this conclusion is same with numerical simulation.

It is well known that the excitation level has an obvious influence on the response characteristics of nonlinear systems. 0.5, 1 and 2 mm are selected as the values of the excitation amplitude *A* is. The excitation frequency changes from 10 to 20 Hz while the value of *d* is 20 mm. It is found that the high-energy interwell oscillations of the ABEH can be induced only when *A* is large enough. The output voltage from high-energy interwell oscillations is much larger than that from intrawell oscillations, as the results shown in Figure 16.

**Figure 17:**
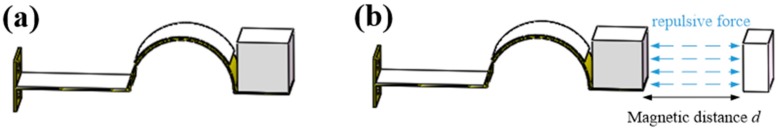
Structural diagram: (**a**) the non-magnet energy harvester; (**b**) the ABEH.

**Figure 18:**
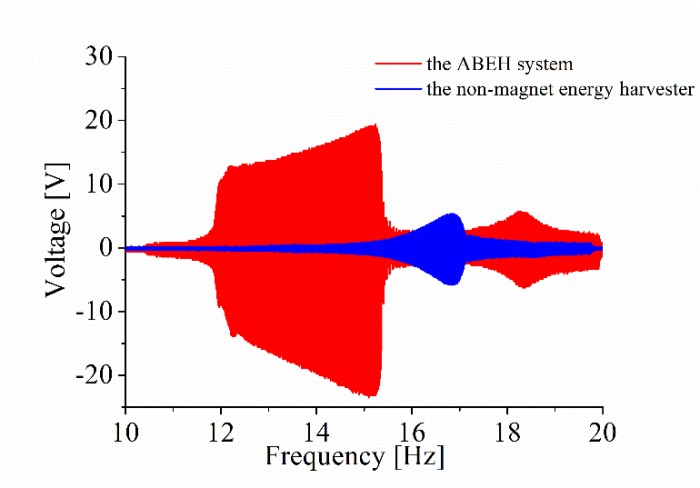
Comparison of energy harvesting performance between the ABEH and the non-magnet energy harvester.

In order to verify the energy harvesting enhancement of the ABEH, the comparison with the non-magnet energy harvester is also provided. Once the external magnet is removed, the ABEH will become a non-magnet harvester, as the structural diagram shown in Figure 17. In experiment, the excitation amplitude *A* is set as 2 mm, and the excitation frequency is ranging from 10 Hz to 20 Hz. The ABEH with *d* = 20 mm is tested and compared with the non-magnet energy harvester, as the output voltage shown in Figure 18. It can be found that the maximum output voltage generated of the non-magnet energy harvester is only about 5 V, and the effective working frequency range is very narrow. On the contrary, the ABEH produces a maximum output voltage of 18 V, which is 3.5 times of that from the non-magnet energy harvester. The nonlinear hardening behavior of the non-magnet energy harvester is caused by the structural nonlinearity. In addition, the effective operating frequency range of the ABEH is more than 3.1 times of that from the non-magnet piezoelectric energy harvester. Therefore, the energy harvesting performance of the ABEH is improved a lot from its non-magnet version. Yang et al. originally designed the linear arc-shaped piezoelectric energy harvester, and they experimentally verified the high-efficiency energy harvesting performance [20]. This work further develops bistable arc-shaped piezoelectric energy harvester to enhance vibration energy harvesting.

## 5. Conclusions

This paper designs an arc-shaped piezoelectric bistable vibration energy harvester (ABEH) based on the arc-shaped cantilever beam and magnetic coupling. By using Lagrangian equation, piezoelectric theory, Kirchhoff’s law, etc., a complete theoretical model of the presented ABEH is built. In simulations, it is found that the quadratic nonlinear stiffness coefficient and the cubic nonlinear stiffness coefficient have obvious influence on the response characteristics and the energy harvesting performance of the ABEH. Meanwhile, the output voltage increases and decreases along with the electromechanical coupling coefficient and the damping, respectively. The distance between the tip magnet and the external magnet plays a key role for determining the nonlinear characteristics of the ABEH. At different excitation frequencies, the optimal load resistance of the ABEH is different. Experimental results verify that the distance between the two magnets influences the energy harvesting performance of the ABEH. More importantly, the ABEH has much better energy harvesting performance than the non-magnet energy harvester. The future work will focus on optimizing strategy for both the shape of the beam, and the magnetic parameters of bistable energy harvester under different excitations for improving vibration energy harvesting performance.
